# Not All Who Stagger Are Drunk: Ocular and Gait Abnormalities in a Young Adult

**DOI:** 10.1016/j.acepjo.2025.100291

**Published:** 2026-01-14

**Authors:** Barry J. Knapp, Jacob Knapp, Lily Kauffman, Hanna Kulbeth, Kean Feyzeau

**Affiliations:** Department of Emergency Medicine, Eastern Virginia Medical School, Norfolk, Virginia, USA

**Keywords:** wernicke, vertical nystagmus, thiamine, ataxia, altered mental status

## 1 Patient Presentation

A 31-year-old woman presented to the emergency department for the third time in 4 weeks with vomiting, unstable gait, and odd behavior. She had a history of chronic nausea and vomiting. She denied any history of alcohol use. Physical examination showed vertical nystagmus (Video), hyperreflexia, ataxia, and an odd affect. Computed tomography and magnetic resonance imaging of the brain were normal. Laboratory results were normal except for a vitamin B1 (thiamine) level of 30 nmol/L (reference range 78-185 nmol/L).

**Video:**
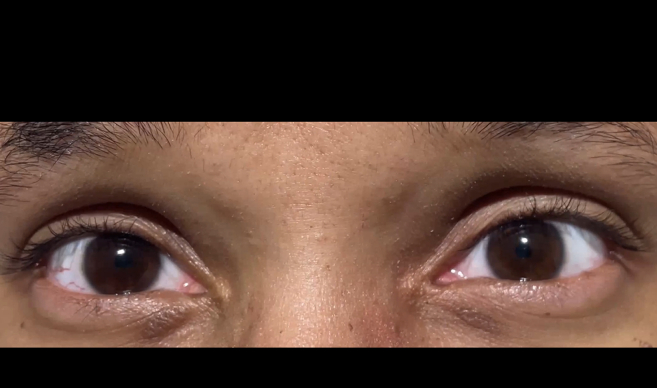
Ocular video demonstrating vertical nystagmus associated with Wernicke encephalopathy.

## 2 Diagnosis

### 2.1 Wernicke Encephalopathy

The patient was diagnosed and treated for Wernicke encephalopathy with thiamine 500 mg every 6 hours for 5 days. She was discharged on hospital day 8 on oral thiamine supplementation.

Wernicke encephalopathy is most commonly a consequence of chronic alcoholism, which impairs thiamine absorption.^1^ However, as in this patient with chronic vomiting, it can also occur with malnutrition, which is uncommon in the United States.

Thiamine plays a vital role in cellular processes, namely the production of ATP. Thiamine deficiency results in metabolic dysfunction that can lead to cell death and the neurologic complications of Wernicke encephalopathy.^1^

The clinical manifestations of Wernicke encephalopathy are described by a triad of ocular dysfunction, ataxia, and altered mental status.^2^ Ocular dysfunction is the principal diagnostic finding. Nystagmus may present vertically—as shown in the Video—or horizontally. Ataxia and altered mental status are frequently observed—with altered mental status present in 90% of patients. The complete triad is found in less than one-third of patients.^2^

Early recognition and prompt thiamine replacement are critical to prevent irreversible cognitive impairment.

## Funding and Support

By *JACEP Open* policy, all authors are required to disclose any and all commercial, financial, and other relationships in any way related to the subject of this article as per ICMJE conflict of interest guidelines (see www.icmje.org). The authors have stated that no such relationships exist.

## Conflict of Interest

All authors have affirmed they have no conflicts of interest to declare.
